# Impact of Intestinal Concentration and Colloidal Structure on the Permeation-Enhancing Efficiency of Sodium Caprate in the Rat

**DOI:** 10.1021/acs.molpharmaceut.1c00724

**Published:** 2021-12-20

**Authors:** Staffan Berg, Lillevi Kärrberg, Denny Suljovic, Frank Seeliger, Magnus Söderberg, Marta Perez-Alcazar, Natalie Van Zuydam, Bertil Abrahamsson, Andreas M Hugerth, Nigel Davies, Christel A. S. Bergström

**Affiliations:** † The Swedish Drug Delivery Center, Department of Pharmacy, Uppsala University, BMC P.O. Box 580, SE-751 23 Uppsala, Sweden; ‡ Advanced Drug Delivery, Pharmaceutical Sciences, R&D, AstraZeneca, 431 83 Gothenburg, Sweden; § Animal Sciences and Technologies, Clinical Pharmacology and Safety Sciences, Biopharmaceuticals R&D, AstraZeneca, 431 83 Gothenburg, Sweden; ∥ Cardiovascular, Renal and Metabolism Safety, Clinical Pharmacology & Safety Sciences, BioPharmaceuticals R&D, AstraZeneca, 431 83 Gothenburg, Sweden; ⊥ Imaging and Data Analytics, Clinical Pharmacology and Safety Sciences, Biopharmaceuticals R&D, AstraZeneca, 431 83 Gothenburg, Sweden; # Data Science and Quantitative Biology, Discovery Sciences, BioPharmaceuticals R&D, AstraZeneca, 431 83 Gothenburg, Sweden; ¶ Oral Product Development, Pharmaceutical Technology & Development, Operations, AstraZeneca, 431 83 Gothenburg, Sweden; ∇ Ferring Pharmaceuticals A/S Global Pharmaceutical R&D, 2300 Copenhagen, Denmark

**Keywords:** sodium caprate, permeation enhancer, FITC-dextran 4000, oral peptide delivery, rat intestinal instillation model

## Abstract

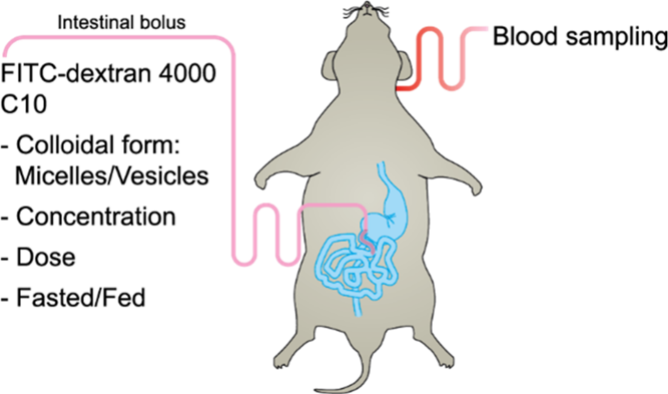

In this work, we set out to better understand how the permeation enhancer sodium caprate (C10) influences the intestinal absorption of macromolecules. FITC-dextran 4000 (FD4) was selected as a model compound and formulated with 50–300 mM C10. Absorption was studied after bolus instillation of liquid formulation to the duodenum of anesthetized rats and intravenously as a reference, whereafter plasma samples were taken and analyzed for FD4 content. It was found that the AUC and *C*_max_ of FD4 increased with increasing C10 concentration. Higher C10 concentrations were associated with an increased and extended absorption but also increased epithelial damage. Depending on the C10 concentration, the intestinal epithelium showed significant recovery already at 60–120 min after administration. At the highest studied C10 concentrations (100 and 300 mM), the absorption of FD4 was not affected by the colloidal structures of C10, with similar absorption obtained when C10 was administered as micelles (pH 8.5) and as vesicles (pH 6.5). In contrast, the FD4 absorption was lower when C10 was administered at 50 mM formulated as micelles as compared to vesicles. Intestinal dilution of C10 and FD4 revealed a trend of decreasing FD4 absorption with increasing intestinal dilution. However, the effect was smaller than that of altering the total administered C10 dose. Absorption was similar when the formulations were prepared in simulated intestinal fluids containing mixed micelles of bile salts and phospholipids and in simple buffer solution. The findings in this study suggest that in order to optimally enhance the absorption of macromolecules, high (≥100 mM) initial intestinal C10 concentrations are likely needed and that both the concentration and total dose of C10 are important parameters.

## Introduction

Sodium caprate (C10) is one of the most studied permeation enhancers for improving oral absorption of compounds with low permeability, such as peptide- and nucleotide-based drugs, across the epithelium of the gastrointestinal tract. C10 is the basis of the gastrointestinal permeation enhancement technology I (GIPET I) platform developed by Merrion Pharmaceuticals, typically containing 500 mg of C10 as an enteric-coated tablet, and has been used in numerous clinical studies in GIPET and other formulations.^1−8^ In these studies, estimated bioavailability values were single-digit with high variability.

Sodium caprate is the sodium salt of the medium-chain fatty acid capric acid, also known as decanoic acid. The p*K*_a_ of the C10 monomer is approximately 4.8. However, being amphiphilic, C10 self-associates in aqueous solution which in turn affects the apparent p*K*_a_, increasing it to around 7 when in the aggregated form, depending on the C10 concentration.^9,10^ The aggregation behavior of C10 in aqueous solution is complex and depends on the fraction of the ionized and unionized species.^10,11^ Under alkaline conditions where C10 predominantly exists in the ionized form (>pH 8.1), micelles are formed. As the pH is reduced, and more C10 exists in the unionized form, lamellar bilayers form, which in dilute solution take the form of vesicles. At pH values below 6, phase separation occurs with C10 precipitating out as an oily phase. The critical aggregation concentration of C10 is also pH-dependent.^10^ At high pH (pH > 8.5), the critical micelle concentration is high (reported to be 50–100 mM^10^). The reason for this is that the charged headgroups cause a high surface charge density and concomitantly high counter-ion binding, which reduces the entropy of the counter ions and so counteracts the micelle formation.^12^ At lower pH values (pH 6.5–7.5), where more C10 is present in the unionized state, the critical vesicle concentration is lowered (reported to be 10–20 mM^10^) as the unionized form helps to lower the surface charge density. The aggregation is made even more complex by the critical aggregation concentration being dependent on both the type of counterion(s) and the ionic strength of the solution. This complex aggregation behavior of C10 likely underlies the wide range of values reported for what is typically referred to as the critical micelle concentration of C10.^4^

C10 transiently and reversibly increases the permeability of the intestinal epithelium by affecting both the paracellular and transcellular absorptive pathways. C10 has been demonstrated to promote paracellular absorption by affecting tight-junction proteins^13−18^ (e.g., claudins, occludin, zonula occludens 1, and tricellulin), opening up the paracellular pathway to enable absorption of macromolecules. C10 is also believed to promote transcellular absorption of hydrophilic macromolecules by transiently increasing membrane fluidity through insertion into the cell-membrane bilayer, thereby perturbating the integrity of the intestinal epithelium.^13,19−21^ It has been proposed that the permeation-enhancing effects are predominantly driven by the ionized, surface active form of C10,^4,22^ but this has not been extensively investigated.

Despite the long history of using and studying C10 for enhancing the permeability of poorly permeable compounds, there is still no consensus on how to best formulate C10 to enable oral absorption of macromolecules such as therapeutic peptides, as illustrated by the reported low single-digit bioavailabilities and high variability. The current understanding is that the macromolecule and enhancer need to be co-delivered to the absorption site (typically the small intestine using an enteric coat), and a sufficiently high local concentration of the enhancer and macromolecule is needed at the intestinal epithelium to enhance the absorption of the macromolecule.^20^ However, rapid dilution of the enhancer and macromolecule in the intestinal lumen, variability in terms of the intestinal fluid-pocket volume and composition, and the variable intestinal motility pattern and transit time are some of the factors that may affect the absorption-enhancing efficiency and thereby cause intra- and interindividual variability in the extent of drug absorption.^23,24^

This work seeks to add new insights into how to best utilize C10 to enable oral absorption of macromolecular drugs such as peptides. Focus was set on the impact of the C10 concentration and its colloidal form, intestinal dilution, and the presence of intestinal components such as bile salt and phospholipids. Fluorescein isothiocyanate-dextran 4000 (FD4; average molecular weight 4000 Da) was selected as a model compound, and its absorption was studied in a rat intestinal instillation model. Absorption was studied for 120 min by analyzing plasma samples for the FD4 content. Histopathological examination was performed at different time points after administration to study the kinetics of the alterations to the intestinal epithelium.

## Materials and Methods

### Materials

The materials were purchased from the following sources: FD4, maleic acid, and sodium hydroxide from Sigma-Aldrich (St. Louis, MO, USA), sodium caprate: Tokyo Chemical Industry (Tokyo, Japan), sodium chloride: Honeywell Fluka (Seelze, Germany), phosphate-buffered saline: Life Technologies Limited (Paisley, UK), tris(hydroxymethyl)aminomethane (TRIS): BDH Laboratory Supplies (Poole, England), sodium taurocholate: Biosynth (Bratislava, Slovakia), phosphatidylcholine from egg (Lipoid E PC S): Lipoid GmbH (Ludwigshafen, Germany), FeSSIF-V2 powder: Biorelevant.com (London, United Kingdom), glucose solution 5% for injection and NaCl 0.9% for injection: Braun (Melsungen, Germany), formaldehyde 4%, 2 M HCl solution, and 2 M NaOH solution: Apotek Produktion & Laboratorier AB (Gothenburg, Sweden). Water was purified with a Millipore Milli-Q Advantage A10 system (Millipore Corporation, Billerica, MA).

### Buffers and Formulations

Tris buffered saline was prepared by dissolving 0.242 g of TRIS and 0.467 g of sodium chloride in Milli-Q water and adjusting the pH to 8.5 and volume to 100 mL. Blank FaSSIF was prepared by dissolving 2.22 g of maleic acid, 1.39 g of sodium hydroxide, and 4.01 g of sodium chloride in Milli-Q water and adjusting the pH to 6.5 and volume to 1000 mL. FaSSIF-V2 was prepared by dissolving 1.61 g of sodium taurocholate and 0.156 g of phosphatidylcholine in blank FaSSIF and adjusting the volume to 1000 mL giving a solution containing 3 mM taurocholate and 0.2 mM phosphatidylcholine. FeSSIF-V2 was prepared according to the manufacturer’s instructions. C10 solutions were prepared at 50 mM, 100 mM, and 300 mM C10 in the various buffers and were pH-adjusted to either 6.5 or 8.5 by addition of 2 M NaOH or 2 M HCl. Blank FaSSIF solutions containing 0–300 mM C10 were stored at room temperature and used within 30 days. The simulated intestinal fluids FaSSIF-V2 and FeSSIF-V2 containing C10 were stored in aliquots at −20 °C.

FD4 was dissolved in the various buffers at a concentration of 12.5 mg/mL, apart from the intestinal dilution studies where concentrations of 6.25 and 37.5 mg/mL were also used. Formulations for absorption studies were prepared the day before the experiment and stored protected from light at +4 °C overnight. Formulations used in the histology study were prepared in an identical manner as the formulations used in the absorption study. The formulations for intravenous (IV) injection were prepared by dissolving FD4 in PBS, pH 7.4, at a concentration of 3 mg/mL, after which the solution was sterile-filtered into autoclaved injection vials.

### Cryo-TEM

Cryo-TEM investigations were performed with a Zeiss Libra 120 transmission electron microscope (Carl Zeiss NTS, Oberkochen, Germany) as previously described.^25^ The microscope was operated at 80 kV and in zero-loss bright-field mode. Digital images were recorded and analyzed using a BioVision Pro-SM Slow Scan CCD camera (Proscan GmbH, Scheuring, Germany) and iTEM software (Olympus Soft Imaging System, GmbH, Münster, Germany). All samples were prepared and treated in the same way as the formulations used in the in vivo studies.

### Dynamic Light Scattering

Dynamic light scattering (DLS) measurements were performed on a Malvern Zetasizer Nano ZSP (Malvern Instruments, Worcestershire, the UK). All samples were analyzed at 25 °C.

### In Vivo Absorption Studies

The studies were approved by the local ethics committee for animal research in Gothenburg, Sweden (ID 1995, approval 5 December 2018). Male Wistar Han rats (Charles River Laboratories, Germany), aged 9–11 weeks, with an average weight of 285 g (SD 24 g, CV 8.4%) were used. The animal-housing room was maintained at 21 °C and 50% RH, with a 12 h light/dark cycle. Upon arrival to the animal facility, the rats received an acclimatization period of one week with food and water ad libitum. Before the experiment, the rats were fasted on grids for 16 h in separate cages with free access to water and a 5% glucose solution.

Anesthesia was induced with 5% isoflurane administered in 2 L/min of compressed air using an Ohmeda Isotec 5 vaporizer (Simtec Engineering, Kungsbacka, Sweden) until effect. Rats were moved to a heated preparatory table, where anesthesia was continued using 3% isoflurane carried in air (0.5 L/min) and oxygen (0.1 L/min). Fur was shaved using Aesculap ISIS electrical clippers (Braun, Melsungen, Germany) from the throat to the lower abdomen. The shaved area was disinfected using a Descutan medicinal sponge (Fresenius Kabi, Bad Homburg, Germany). Artelac eyedrops (Bausch and Lomb, London, UK) were applied to prevent drying of the eyes. Each rat was wrapped in Glad Press N Seal plastic foil (The Glad Products Company, Oakland, USA) and transferred to a preheated operating table where anesthesia was continued throughout the entire study using 3% isoflurane. The left carotid artery was partially exposed by a small incision in the supraclavicular region, approximately 2 cm long. A polyurethane catheter with a rounded tip (Instech Laboratories, Plymouth Meeting, PA, USA) was placed in the carotid artery and secured with nylon sutures. Thereafter, a midline incision was made in the abdomen. The bile duct was located and catheterized with an Intramedic PE10 polyethylene tubing (Beckton Dickinson, Sparks, MD, USA), which was then secured with nylon sutures. The stomach was punctured approximately 1 cm proximal to the pyloric sphincter using a 20G needle (Braun, Melsungen, Germany). A soft polyurethane catheter with a rounded tip (identical to the carotid catheter) was inserted into the intestine via the gastric incision so that the tip of the catheter was positioned approximately 4 cm distal to the pylorus. The intestinal catheter was secured to the stomach using a nylon suture, and a ligature was placed at the pylorus to prevent backflow of the formulation into the stomach and transit of the gastric content into the intestine. A thermometer was placed in the abdominal cavity, and the abdomen was closed with stitches. The thermometer was coupled to a heating lamp via a thermostat set to keep the animal at 37 °C. The surgery was followed by a recovery period of 30 min to allow the animal to regain normal blood pressure and temperature. The carotid catheter was connected to a three-way valve to allow for blood sampling into K2 EDTA-coated microcentrifuge tubes (Sarstedt, Numbrecht, Germany). The three-way valve connects to a four-way junction, allowing for the following: (a) a continuous catheter flow (10 μL/min) of 20.6 mM sodium citrate in 0.9% saline solution to prevent clotting using a CMA100 microinjection pump (Carnegie Medicin, Stockholm, Sweden); (b) measurement of blood pressure using a bespoke blood pressure measurement system developed in-house at AstraZeneca R&D Gothenburg; and (c) connection of a syringe for drawing blood through the carotid catheter during blood sampling.

The formulations to be administered were equilibrated to room temperature, under magnet stirring, for 2 h before administration. Intestinal bolus administrations were performed over ∼5 s via the intestinal catheter. The dose volume was 0.8 mL, except in the intestinal dilution study where dose volumes were 0.27, 0.8, and 1.6 mL. Five replicates were performed for each administration group. The administered FD4 dose was 10 mg in 50 mM (7.8 mg), 100 mM (16 mg), or 300 mM (47 mg) of C10 adjusted to pH 6.5 or 8.5. In the intestinal dilution study, all animals received 10 mg of FD4 and 16 mg of C10 in a dose volume of 0.27 mL (300 mM C10), 0.8 mL (100 mM C10), or 1.6 mL (50 mM C10), all adjusted to pH 6.5. Blood samples (200 μL) were drawn before administration and at 5, 10, 20, 30, 45, 60, and 120 min post administration. Blood plasma was separated by immediately centrifuging the blood samples at +4 °C and 10,000 g for 4 min in an Eppendorf 5415 R centrifuge (Eppendorf, Hamburg, Germany). Plasma samples were transferred to new tubes and stored at −80 °C until analysis. Animals that did not maintain an average blood pressure of 70 mmHg or higher during the administration and blood-sampling period were excluded to secure representative physiological conditions.

The IV-administered animals were handled and prepared in the same way as the animals receiving intestinal administration up until placement of the carotid catheter. As no abdominal surgery was performed, a thermometer was introduced rectally. A recovery period of 30 min was allowed before the IV administration of 5 mg/kg FD4 into the tail vein (*n* = 6).

### Histopathology Study

For histopathological examination of the intestine, the animals were handled and prepared in the same way as the animals in the absorption study. 0.8 mL of the pH 6.5 formulations in blank FaSSIF, as those used in the absorption studies, was administered intraduodenally (ID), in the sham-operated group, no formulation was administered. At 10, 30, 60, and 120 min after administration, the abdomen was opened, and the animal was euthanized. The tip of the administration catheter in the duodenum was located, and an intestinal segment, approximately 6 cm long, was excised. The intestinal segment was carefully rinsed with 5 mL of 4% formaldehyde in PBS solution, placed on a filter paper, and stored in the same fixation solution at room temperature until further preparation. The intestinal segments analyzed were located approximately 2–4 cm distal to the tip of the administration catheter, corresponding to approximately 6–8 cm distal to the pylorus. Four transversal 1 mm serial sections and one longitudinal intestinal section were embedded in paraffin and finally prepared in 4 μm thick sections. Intestine transversal and longitudinal sections were stained with hematoxylin and eosin (H&E, morphology characterization). All histological slides were blinded, scanned using a scanner (Aperio ScanScope XT, Leica Biosystems, Germany), and examined by an experienced pathologist. Histopathological analysis was performed by comparing treatment groups versus controls. Any findings were semiquantitatively scored for apical enterocyte loss and villus blunting from 0 to 4 as no findings (0), minimal (1), mild (2), moderate (3), or severe (4).

### pH Measurement of Unabsorbed Formulation

Animals were handled and prepared in the same way as the animals in the absorption study. 0.8 mL of a formulation containing 300 mM C10 and 12.5 mg/mL FD4 in blank FaSSIF with a pH of 8.5 was administered as a bolus into the duodenum. At predetermined timepoints after administration, the animal was euthanized, and an intestinal segment containing the formulation was excised. The content of the intestinal segment was immediately collected into an Eppendorf tube, and the pH of the intestinal content was measured without delay on a freshly calibrated pH meter (S20 SevenEasy Mettler-Toledo, Ohio, USA). The location of the formulation was possible to identify due to the yellow color of the formulation. The intestinal content was collected at 4, 8, 14, and 20 min post administration. The experiment was performed in duplicate.

### Bioanalysis of Plasma Samples

#### FITC-Dextran Quantification from Blood Plasma

The plasma samples were thawed, and 80 μL of plasma was transferred to a 96-well plate (Thermo Fisher Scientific, Waltham, USA). For quantification, two sets of calibration standards with a known concentration of FD4 in blank plasma were added to the 96-well plate. Stock solutions of FD4 were prepared in PBS, pH 7.4, and stored at −80 °C. Calibration samples were prepared by a 20-fold dilution of stock solutions in blank plasma. The plates were analyzed for fluorescence emission using a multimode plate reader (PerkinElmer, Waltham, USA), with excitation at λ494 nm and emission at λ518 nm. Study samples were quantified with a four-parameter logistic regression against the response from the calibration standards.

#### C10 Quantification from Blood Plasma

25 μL of plasma was mixed with 25 μL of the internal standard (deuterated capric acid, 2.5 μg/mL) in acetonitrile and 50 μL of 2,2-dimethoxypropane and vortexed. 75 μL of a 1.3 M boron trifluoride-methanol solution was added and incubated at 50 °C for 30 min. 400 μL of *n*-hexane was added and vortexed to homogenize. 250 μL of the upper organic phase was transferred to a vial with a silanized glass insert and analyzed by gas chromatography with mass spectrometric detection (GC MS/MS) on an Agilent 7890B system (Agilent Technologies, Paolo Alto, CA, USA) coupled to an Agilent triple quad mass spectrometer 7000C equipped with an electron ionization source. Separation was achieved over a DB-FFAP column, 30 m × 250 μm i.d. (J&W Scientific, Folsom, CA, USA). Helium was used as carrier gas at a constant flow of 1.4 mL/min. The injection volume was 1 μL. The initial oven temperature was set to 60 °C for 0.5 min and thereafter increased to 170 °C at a rate of 20 °C/min, after which the temperature was increased to 250 °C at a rate of 50 °C/min and held for 0.5 min. The total run time was 8.1 min. The lower limit of quantification of the assay was 0.5 μg/mL.

### Pharmacokinetic Analysis

The FD4 plasma concentration versus time data was analyzed using noncompartmental analysis in Phoenix WinNonlin version 8.2 (Certara USA, Inc., Princeton, NJ). The area under the individual plasma concentration–time curve (AUC) from time zero to 120 min (AUC_0–120_) was calculated with the linear trapezoidal method. Bioavailability from zero to 120 min after intraduodenal administration was calculated according to
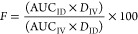
where *F* is bioavailability in percent, AUC is the area under the plasma concentration–time curve from time zero to 120 min, and *D* is the administered dose, ID or IV. Numerical deconvolution was used to estimate the cumulative fraction absorbed over time as described by Langenbucher.^26^ The average, dose-normalized plasma concentrations following the intravenous administration of FD4 were used as weighting function *W*(*t*), and dose-normalized plasma concentrations following duodenal administrations were assigned to the response function *R*(*t*). The correlation of the weighting and response functions depends on the input and can be described according to:
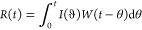


The input function *I*(*t*) was calculated from numerical deconvolution according to:
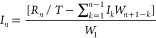
Where *I*_*n*_ is the fraction absorbed, *T* is the time interval, and, for example, *I*_8_ and *W*_8_ are the average input rate and weight, respectively, in the time interval (*T*_7_, *T*_8_). The deconvolution was carried out in GI-Sim^27−29^ version 5.4 with the time step set to 0.5 min. The mean cumulative input function for each administration group is plotted which corresponds to the fraction absorbed.

### Statistical Analysis

Statistical analysis was performed in R (version 3.6.0, R Foundation for Statistical Computing, Vienna, Austria). A two-way ANOVA was used to analyze FD4 *F* and *C*_max_ values in the pH and buffer studies, whereas a one-way ANOVA was used in the dilution study. A sandwich operator was applied to account for heteroscedasticity, and *p* values were adjusted using the Tukey method to adjust for multiple comparisons. To compare the *C*_max_ values of C10, a two-sample *t*-test was selected. A threshold of *p* < 0.05 was used in all cases to declare that differences in means between dose groups were statistically significant. Results are presented as mean ± SD unless otherwise stated.

## Results

### Physical Characterization of Colloidal C10 Solutions

**Figure 1:**
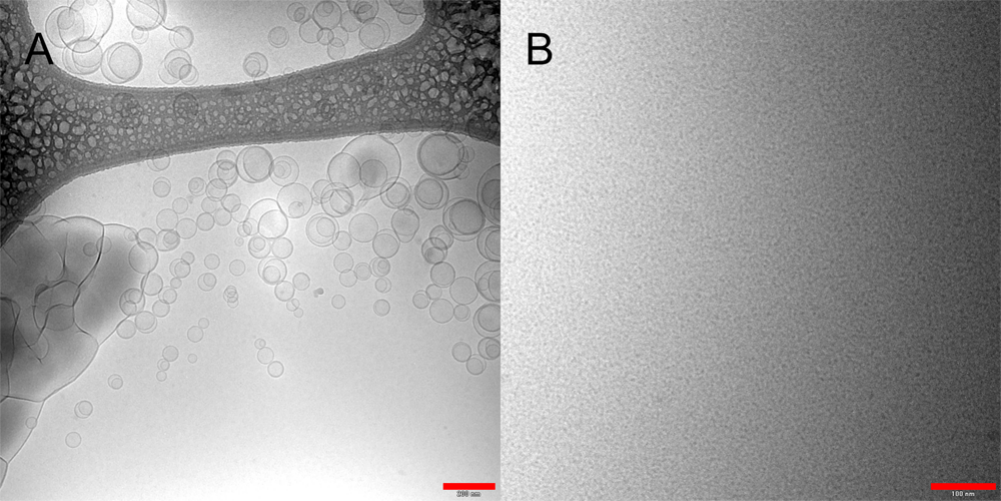
Cryo-TEM images of sodium caprate solutions in blank FaSSIF. (A) 100 mM C10 pH 6.5. Vesicles varying in size from 50 to 200 nm are present in the sample together with larger, more aggregated structures. (B) 300 mM C10 pH 8.5. A homogenous sample of spherical micelles smaller than 5 nm. Scale bar: 200 nm in (A) and 100 nm in (B).

At pH 6.5, the C10 solutions were optically turbid, and cryo-TEM imaging of 100 mM in blank FaSSIF showed vesicular structures ranging from 50 to several hundred nanometers in diameter (Figure 1A). At 300 mM, the vesicular structures of C10 appeared larger. Analysis with DLS of a 50 mM sample in the same buffer showed a broad size distribution with an average size of 280 nm and a polydispersity index of 0.43 (a lower concentration of 50 mM was used for DLS due to obscuration of the signal at higher concentrations).

At pH 8.5, all the C10 solutions were optically clear. Cryo-TEM imaging showed only spherical micelles with an estimated size of less than 5 nm (Figure 1B). DLS indicated a single narrow peak with an average size of 3.0 nm and a polydispersity index of 0.12.

Visual inspection, cryo-TEM imaging, and DLS measurements all support the conclusion that C10 forms micelles at pH 8.5 and vesicles and other larger structures at pH 6.5. This is in agreement with previously published data.^10^

### IV Administration of FD4

The average plasma concentration–time profile following the intravenous administration of 5 mg/kg FD4 is shown in Figure S1 in the Supporting Information. The estimated pharmacokinetic parameters from the noncompartmental analysis are presented in Table 1. The results are in agreement with previously published values for FD4.^30^

**Table 1:** Pharmacokinetic Parameters for FD4 Following Intravenous Administration to Six Rats^a^

parameter	mean ± SD (CV %)
Kel (min^–1^)	0.0257 ± 0.0034 (13)
*t*_1/2_ (min)	27.4 ± 3.6 (13)
MRT (min)	29.4 ± 3.5 (12)
Cl (mL/min/kg)	6.82 ± 0.48 (7)
*V*_z_ (mL/kg)	270 ± 47 (17)
*V*_ss_ (mL/kg)	202 ± 30 (15)
AUC_0–120_ (min μg/mL)	733 ± 60 (8.2)

a Kel: elimination rate constant of the terminal phase, *t*_1/2_: terminal half-life, Cl: total body clearance, MRT: mean residence time, *V*_z_: volume of distribution based on the terminal phase, *V*_ss_: estimated volume of distribution at steady-state, and AUC_0–120_: area under the plasma concentration–time curve from 0 to 120 min.

### In Vivo Absorption of FD4 and C10

#### Impact of C10 Concentration and Colloidal Form on the Absorption of FD4

**Figure 2:**
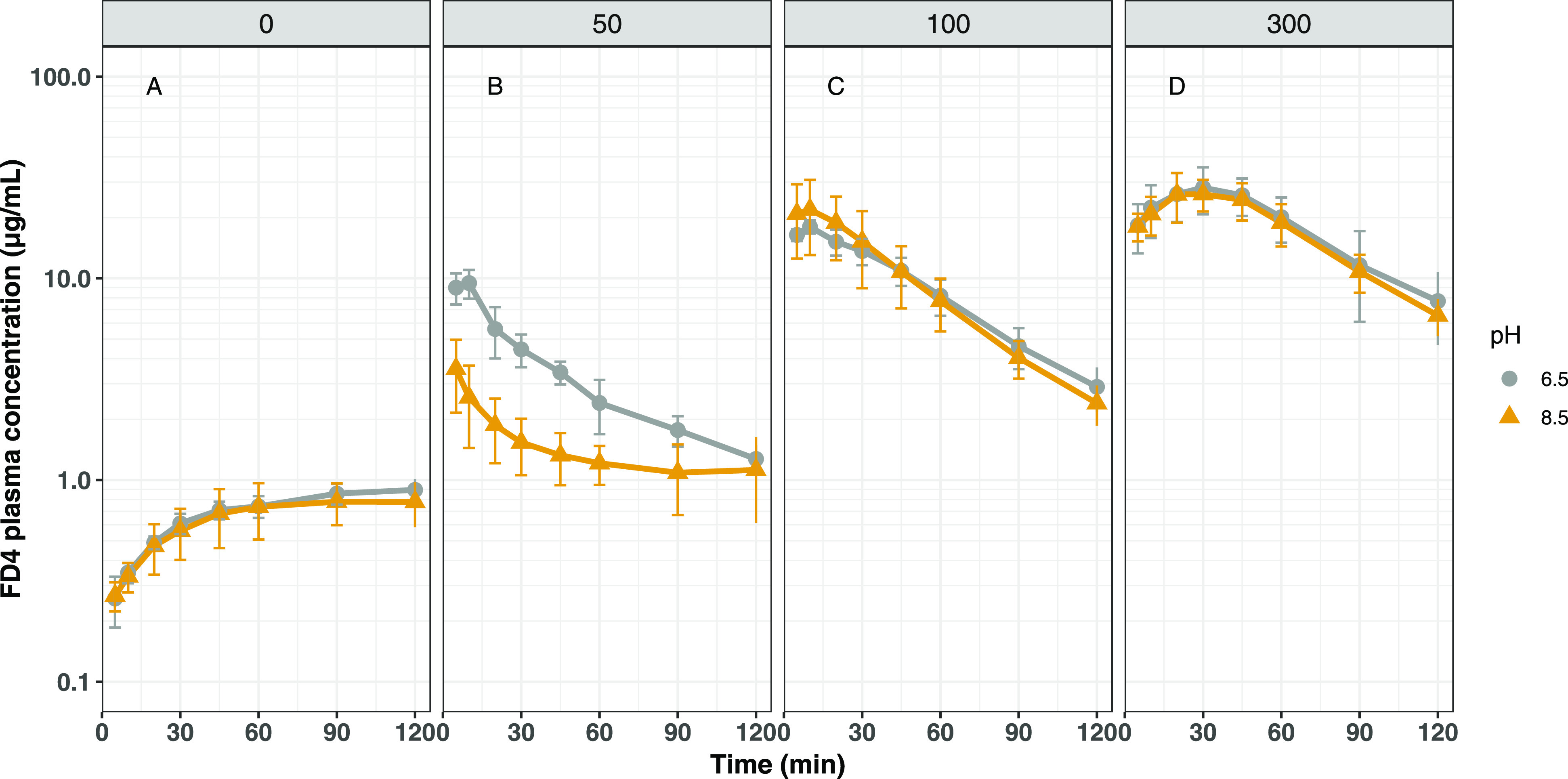
Mean (±SD) FD4 plasma concentration–time profiles after intraduodenal bolus administrations to anesthetized rats (*n* = 5/group). The formulations contained 0 (A), 50 (B), 100 (C), or 300 mM (D) C10 in blank FaSSIF and were pH-adjusted to either pH 6.5 (gray) or 8.5 (yellow). The administration volume was 0.8 mL in all cases corresponding to a C10 dose of 0/7.8/16/47 mg for (A)/(B)/(C)/(D).

The absorption of FD4, in the absence of C10, following the bolus administration of a buffered solution (pH 6.5 or 8.5) into the upper small intestine of rats is shown in Figure 2A. As can be seen, some absorption into the systemic circulation is observed over the 2 h period of the study resulting in an estimated bioavailability of 1.5–1.6%, which appears independent of the pH of the administered formulation. The absorption of FD4 in the presence of different concentrations of C10 is shown in Figure 2B–D. As can be seen from the absorption profiles, absorption of FD4 is enhanced in the presence of C10, consistent with its reported function as a permeation enhancer for macromolecules, and is concentration-dependent (Table 2). At 50 mM C10, peak plasma levels are at least four-fold compared to those achieved in the absence of C10, and peak levels are also observed at the earliest time points of sampling (5–10 min) compared to what appears to be a more slow and continuous absorption of FD4 in the absence of C10. Interestingly, higher bioavailability (two-fold, *p* < 0.001) and peak plasma levels of FD4 (two-fold, *p* < 0.001) were observed when administered at pH 6.5 compared to at pH 8.5 at this C10 concentration, presumably a result of the different colloidal forms of C10 presented to the intestinal epithelium. Interestingly however, at C10 concentrations of 100 and 300 mM, no effect of the pH/colloidal form of C10 on the absorption of FD4 is observed, and the absorption profiles of FD4 are superimposable (Figure 2C,D). As can also be seen from the absorption profiles, increasing the concentration of C10 extends the absorption window with peak plasma levels being observed typically at 5–10 min for 50 mM C10, at around 10 min for 100 mM of C10, and approximately 30 min for 300 mM C10 (independent of the pH of the formulation). Estimated pharmacokinetic parameters when administered in the different formulations are reported in Table 2.

**Table 2:** Pharmacokinetic Parameters of FD4 after Intraduodenal Administration of Different Formulations to Fasted Rats^a^

C10 conc., mM	formulation pH	AUC_0–120_, min μg/mL	*F*, %	*C*_max_, μg/mL	*t*_max_, min
0	6.5	82.8 ± 7.6 (9.2)	1.6 ± 0.15 (9.2)	0.91 ± 0.12 (13)	90 (90/120)
0	8.5	77.5 ± 19 (24)	1.5 ± 0.34 (23)	0.84 ± 0.23 (28)	90 (60/120)
50	6.5	405 ± 60 (15)	7.9 ± 1.2 (15)	9.5 ± 1.6 (17)	10 (5/10)
50	8.5	172 ± 51 (29)	3.3 ± 0.97 (29)	3.6 ± 1.4 (39)	5 (5/5)
100	6.5	1070 ± 160 (15)	21 ± 3.3 (16)	18 ± 1.3 (7.4)	10 (10/10)
100	8.5	1140 ± 380 (33)	22 ± 7.2 (32)	22 ± 8.9 (40)	10 (5/10)
300	6.5	2180 ± 540 (25)	44 ± 11 (26)	28 ± 6.7 (23)	30 (30/45)
300	8.5	2050 ± 280 (14)	40 ± 5.6 (14)	29 ± 6.8 (24)	30 (20/45)

a AUC, *F*, and *C*_max_ are reported as mean ± SD (CV %), *t*_max_ as median (min/max), *n* = 5/group. All formulations were prepared in blank FaSSIF.

**Figure 3:**
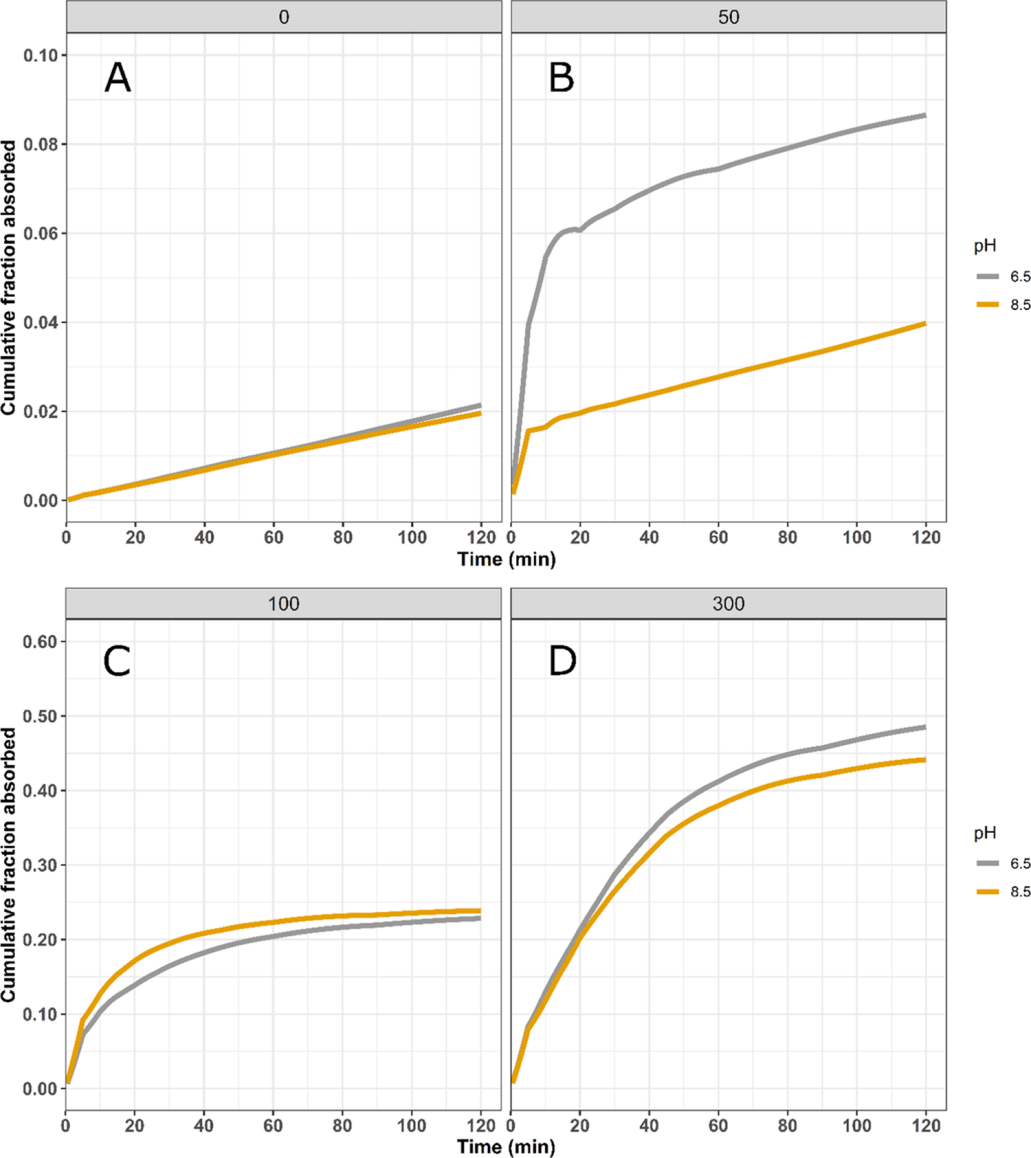
Cumulative average FD4 fraction absorbed vs time (*n* = 5/group). Formulations contained 0 (A), 50 (B), 100 (C), or 300 mM (D) C10 in blank FaSSIF and pH-adjusted to 6.5 or 8.5.

The cumulative fraction of FD4 absorbed with respect to time was estimated by deconvolution of the plasma concentration–time profiles. The absorption profiles for the various treatments are reported in Figure 3. In the absence of C10, the absorption rate of FD4 appeared constant over the 2 h period of the study and was similar at both pH levels investigated. For formulations containing 50 mM C10, the majority of the FD4 absorption took place during the first 5 or 10 min for formulations with a pH of 8.5 and 6.5, respectively. After the initial absorption window, the FD4 absorption rate decreased to a rate similar to that observed for formulations not containing C10. For formulations containing 100 and 300 mM C10, again biphasic absorption profiles were observed with an initial rapid absorption phase followed by a phase with a lower absorption rate. Here, however, the second phase displayed a higher absorption rate than that observed in the absence of C10. For these two C10 concentrations, the pH of the formulation had negligible effect on the absorption profiles. The absorption window, where increased FD4 was absorbed compared to formulations not containing C10, however, was observed to be longer as the C10 concentration was increased; approximately 30 and 60 min for 100 and 300 mM C10, respectively.

#### Absorption of C10

**Figure 4:**
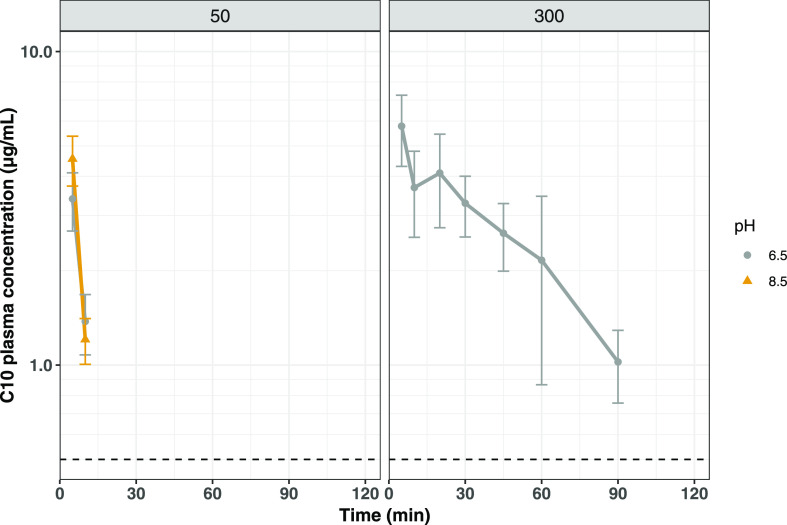
Mean (±SD) C10 plasma concentration–time profiles after intraduodenal bolus administrations to anesthetized rats (*n* = 5/group). The formulations contained 50 mM C10 pH 6.5, 50 mM C10 pH 8.5, and 300 mM C10 pH 6.5 in blank FaSSIF. The dashed line indicates the lower limit of quantification.

Plasma samples following the administration of 50 mM C10 at pH 6.5 and pH 8.5 were analyzed for C10 content in order to better understand the difference in absorption profiles of FD4 when formulated at the two different pH levels. Plasma samples following the administration of 300 mM C10 formulated at pH 6.5 were also analyzed. The results from the C10 bioanalysis are presented in Figure 4 and Table 3, and individual profiles are presented in Figure S2 of the Supporting Information. As can be seen, C10 was in all cases rapidly absorbed with a *t*_max_ being observed at the first sampling time of 5 min. The absorbed C10 quickly fell below the limit of quantification (0.5 μg/mL) when the administered concentration was 50 mM, whereas for the higher concentration, the apparent elimination rate was lower, likely a result of prolonged C10 absorption. For administrations of 50 mM C10, a higher *C*_max_ was achieved when C10 was formulated at pH 8.5 compared to when formulated at pH 6.5 (4.5 ± 0.82 vs 3.4 ± 0.71 μg/mL; *p* = 0.035), possibly indicating that C10 is more rapidly absorbed when presented in the micellar form. The calculated AUC for exposure appeared dose-proportional and also pH-independent being 35.3 ± 21 min μg/mL for 50 mM C10 at pH 6.5, 37.6 ± 20 min μg/mL for C10 at pH 8.5, and 264 ± 67 min μg/mL for 300 mM C10 at pH 6.5. These results show that C10 is rapidly and likely completely absorbed following instillation of solutions into the upper small intestine of rats.

**Table 3:** C10 Pharmacokinetic Parameters after Intraduodenal Administration to Rats^a^

C10 concentration, Mm	formulation pH	AUC_0–120_, min μg/mL	*C*_max_, μg/mL	*t*_max_, min
50	6.5	35.3 ± 21 (59)	3.4 ± 0.71 (21)	5 (5/5)
50	8.5	37.6 ± 20 (54)	4.5 ± 0.82 (18)	5 (5/5)
300	6.5	264 ± 67 (25)	5.8 ± 1.5 (26)	5 (5/5)

a AUC and *C*_max_ are reported as mean ± SD (CV %), *t*_max_ as median (min/max), *n* = 5/group.

### Determination of pH of Nonabsorbed Formulation

**Figure 5:**
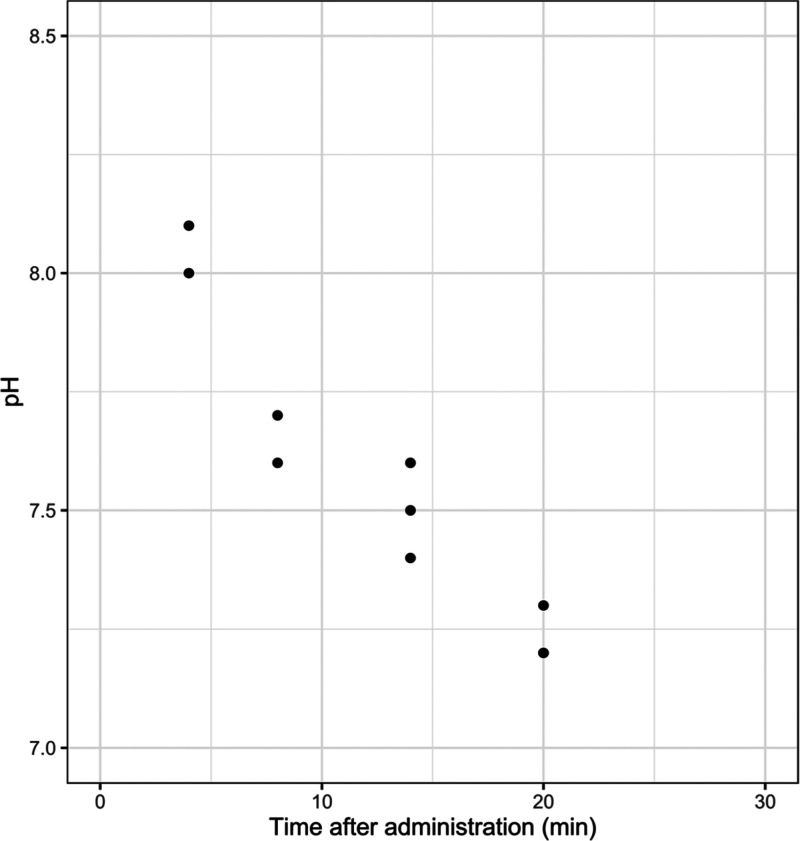
pH of the intestinal lumen content vs time following the intestinal bolus administration of 300 mM C10 in blank FaSSIF. The pH of the administered formulations was 8.5 (*n* = 2–3/time point).

The pH of the residual formulation in the small intestine following the administration of 0.8 mL of a 300 mM C10 formulation containing 12.5 mg/mL FD4 adjusted to 8.5 as a function of time, as shown in Figure 5. The pH of the residual formulation and intestinal contents reduces rapidly and within 20 min had dropped by over one pH unit.

### Histopathology

**Figure 6:**
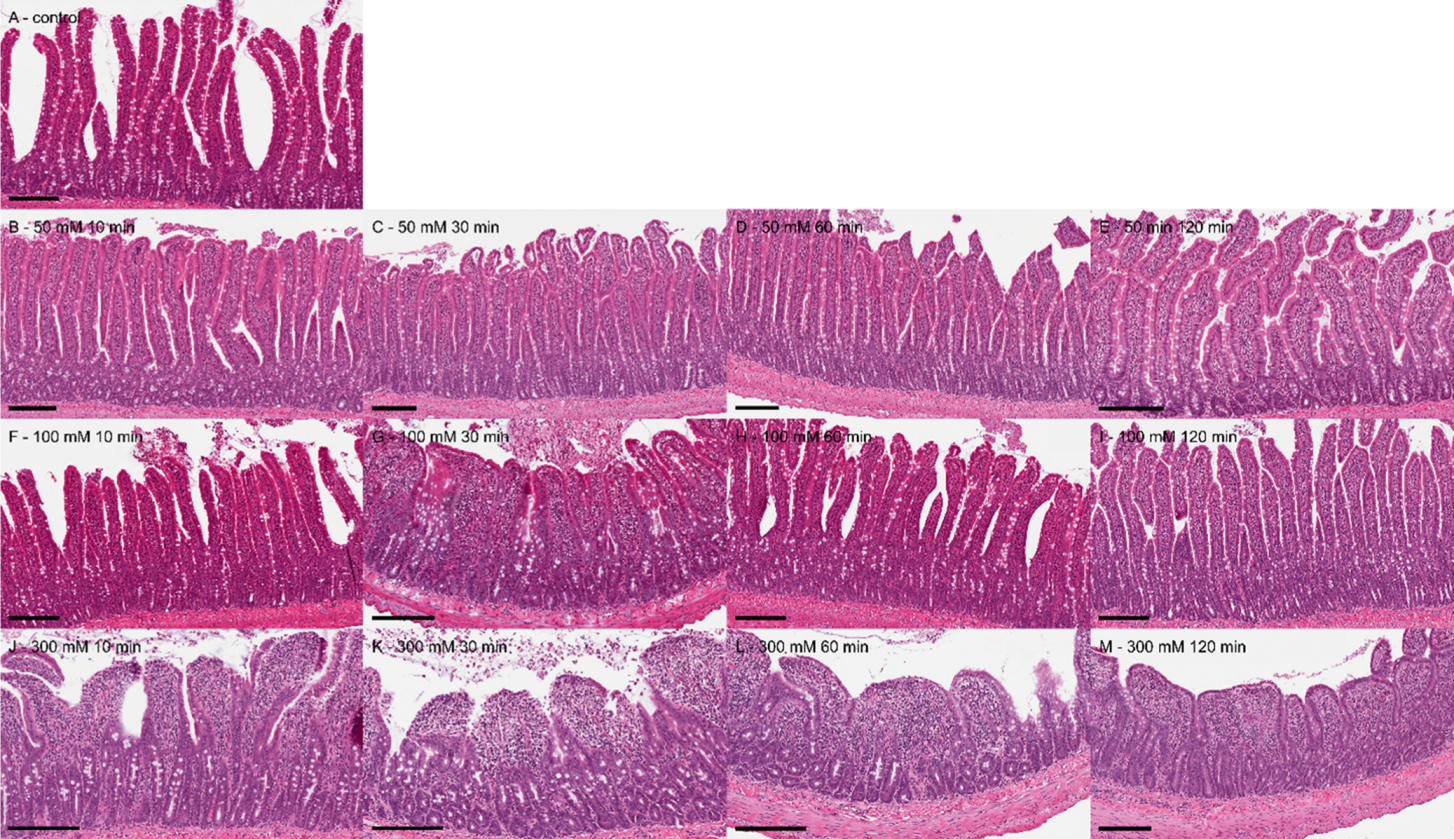
Representative photomicrographs of intestinal mucosa at different concentrations of C10 examined at different timepoints after administration. Scale bar indicates 200 μm.

Representative images of the intestinal mucosa exposed to different concentrations of C10 and at different time points after administration are presented in Figure 6. The sham group, where no formulation was administered, showed normal histology with long, separated villi displaying an intact epithelium (Figure 6A). The control group, receiving FD4 in blank FaSSIF with no C10, also displayed normal histology (images not shown), verifying that the vehicle solution and concentration of FD4 used did not affect the integrity of the mucosa.

Histological images of the intestine following exposure to 50 mM C10 are shown in Figure 6B–E. Here, the intestinal epithelium is largely intact with some detachment of enterocytes from the apical villi observed at 10 and 30 min (Figure 6B,C, respectively) after administration. The epithelium had fully recovered 60 min (Figure 6D) post administration. Administration of 100 mM C10 resulted in more extensive erosion of the enterocyte layer at the tip of the villi at 10 and 30 min (Figure 6F,G, respectively) after dose administration. At 30 min, a blunting of the villi was observed in addition to the epithelial changes. Full recovery was again observed 60 min (Figure 6H) post administration. After exposure to 300 mM C10, erosion of the enterocyte layer was evident at 10–60 min (Figure 6J–L) after administration, ranging from severe at 10 min to moderate at 30 min to mild at 60 min. At 120 min (Figure 6M) after administration, the enterocyte layer again showed recovery with the epithelium covering the villus submucosa, although mucosal remodeling with a thin surface epithelium and blunting of villi was still evident. Table 4 summarizes the results of the histological findings and severity scoring of the observations relating to apical enterocyte loss and villus blunting.

**Table 4:** Results from the Histopathology Evaluation

		histology score	
C10 concentration (mM)	timepoint after dose (min)	apical enterocyte loss (0–4)	villus blunting (0–4)
50	10	1	0
50	30	1	0
50	60	0	0
50	120	0	0
100	10	2	0
100	30	1	2
100	60	0	0
100	120	0	0
300	10	4	2
300	30	3	3
300	60	2	3
300	120	0	2
0 (vehicle)	10	0	0
0 (vehicle)	120	0	0
Sham	10	0	0
Sham	120	0	0

Rats were administered formulations containing different concentrations of C10 dissolved in blank FaSSIF adjusted to pH 6.5. Sham animals underwent the same handling and treatment, including surgery, but no formulation was administered.

### Intestinal Dilution Study

**Figure 7:**
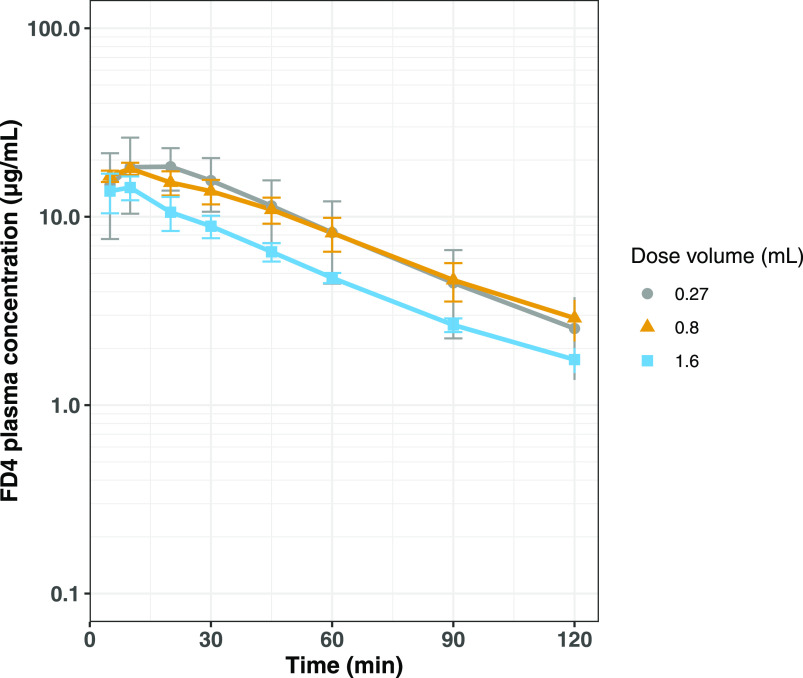
Average (±SD) FD4 plasma concentration–time profiles after intraduodenal bolus administrations to anesthetized rats (*n* = 5/group). The formulations contained 10 mg of FD4 and 16 mg of C10 dissolved in 0.27 (gray circles), 0.80 (orange triangles), or 1.6 mL of (blue squares) of blank FaSSIF. All formulations were adjusted to pH 6.5.

In the absorption studies described above, a constant concentration of FD4 (12.5 mg/mL) was administered as a bolus into the upper small intestine of rats together with various concentrations of C10 formulated at pH 6.5 or 8.5. To investigate the possible implications of a single-unit dosage form, such as a tablet, containing C10 as a permeation enhancer depositing and dissolving in intestinal fluid pockets of different volumes, we explored the effect of administering solutions having the same ratio of FD4 to C10 but having different concentrations of these components. The aim was to replicate the scenario of a dosage form dissolving in fluid pockets with different volumes. 10 mg of FD4 and 16 mg of C10 were dissolved in blank FaSSIF and administered at three different volumes: 0.27, 0.8 (standard volume used in this work), and 1.6 mL. The resulting FD4 plasma concentration profiles are presented in Figure 7. Rather similar absorption profiles were observed for formulations administered at volumes of 0.8 and 0.27 mL representing C10 concentrations of 100 and 300 mM, respectively. Administration of the same dose of FD4 and C10 but in a larger volume of 1.6 mL (equating to a C10 concentration of 50 mM) resulted in less FD4 absorption with exposure (AUC_0–120_) being 703 ± 67 min μg/mL, compared to 1070 ± 160 min μg/mL when administered in 0.8 mL and 1120 ± 390 min μg/mL when administered in 0.27 mL (values presented as mean ± SD), however not significant at *p* = 0.059. The pharmacokinetic parameters are presented in the Supporting Information (Table S1).

### Effect of Vehicle Composition

**Figure 8:**
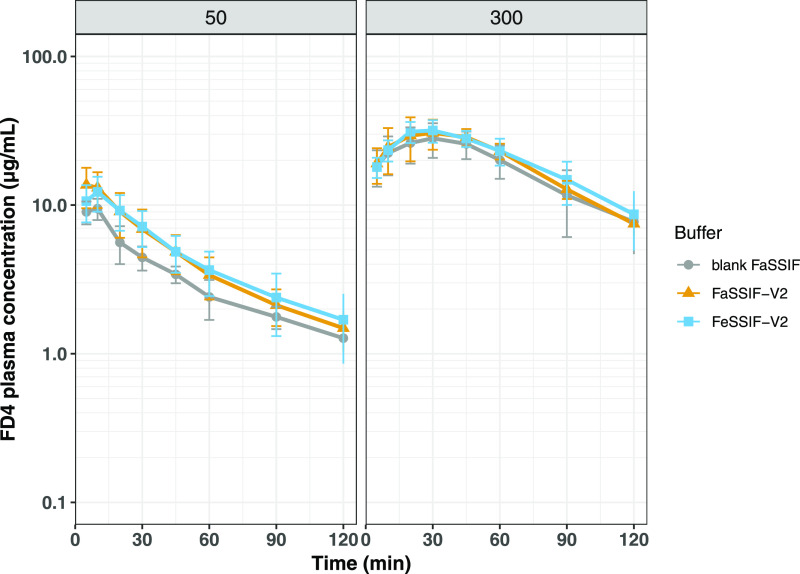
Mean (±SD) FD4 plasma concentration–time profiles after intraduodenal bolus administrations to anesthetized rats (*n* = 5/group). The formulations contained 50 or 300 mM C10 dissolved in either buffer alone (blank FaSSIF) or the fasted- and fed state-simulated intestinal fluids FaSSIF-V2 and FeSSIF-V2. All formulations were adjusted to pH 6.5.

To simulate the fasted- and fed-state conditions in the intestine, formulations having a C10 concentration of 50 and 300 mM were formulated in blank FaSSIF (buffer only) or in FaSSIF-V2 or FeSSIF-V2, all at pH 6.5. The average FD4 plasma concentration–time profiles after intraduodenal administration of these formulations are presented in Figure 8 with the corresponding PK parameters in Table S2 (Supporting Information). No difference in exposure or *C*_max_ could be seen between the formulations prepared in buffer alone (blank FaSSIF) and the fasted state-simulated intestinal fluid (FaSSIF-V2) and fed state-simulated intestinal fluid (FeSSIF-V2) at either of the two studied C10 concentrations. If anything, absorption was slightly enhanced when phospholipids and bile salt were included in the formulation containing the lower amount of C10 (50 mM), although not statistically significant (*p* > 0.15 for bioavailability and *p* > 0.20 for *C*_max_).

## Discussion

In order to study if there is a difference in the permeation-enhancing efficiency of C10 when it is delivered in its ionized micellar form compared to its partially ionized form that favors the formation of vesicles, C10 was formulated at pH 8.5 or 6.5 and instilled into the small intestine of the rat as a bolus. The results showed no difference between the two pH levels at C10 concentrations of 100 and 300 mM. At 50 mM C10, however, the absorption of FD4 was higher at pH 6.5 than at pH 8.5. Selected dose groups were chosen for quantification of C10 in plasma in order to understand the reason behind the observed differences. Higher C10 *C*_max_ values were obtained when C10 was formulated at pH 8.5 compared to when formulated at pH 6.5. The higher *C*_max_ suggests that C10 was absorbed quicker when delivered at pH 8.5, as the same dose was administered in both cases. The higher rate of absorption for the micellar species might be due to their smaller colloidal size and concomitantly higher diffusivity in the intestinal lumen and through the mucus layer, compared to the larger vesicles present at pH 6.5. As C10 is more rapidly absorbed at pH 8.5, it suggests that it is more rapidly removed from the intestine and intestinal epithelium where it exerts its permeation-enhancing effect. This hypothesis is consistent with the deconvolution results that suggest that absorption took place during the first 5 min when formulated as micelles and for the first 10 min when formulated as vesicles at 50 mM C10 (Figure 3B). Our results indicate that it may, therefore, be beneficial to prolong the residence time of C10 in the intestinal lumen or, alternatively, to prolong its release from the dosage form, in order to extend the absorption window time-wise. It must be noted, however, that the effect of increasing the C10 concentration, and hence also the dose, had a much greater impact on the absorption of FD4 than the pH of the formulation had (Table 2).

One possible limitation of the pH study, where the two different colloidal forms of C10 were compared, is that the intestine may rapidly neutralize the formulation with a pH of 8.5 as it strives toward its native pH of approximately 6.0–7.5.^31−34^ The results could get convoluted if the neutralization of the administered formulation happens before the majority of C10 is absorbed. To study this, we administered 300 mM C10 adjusted to pH 8.5 and excised the intestinal segments containing the nonabsorbed formulation at different time points after administration. The results showed that the intestinal content had a pH of 8.0 or higher at 4 min after administration. As C10 forms micelles above pH 8.0–8.1^10^ and the C10 pharmacokinetic analysis showed that the majority of C10 was absorbed during the first 5 min (Table 3), we conclude that it is likely that the majority of C10 was absorbed when C10 was still present as micelles. Beyond 4 min after administration, it is possible that the small amounts of remaining C10 could have formed vesicles in the lumen, provided that the concentration would still be above the critical aggregation concentration. 20 min after administration, the intestinal pH had returned to the normal intestinal pH of 7.3.

The results of the histological evaluations support the observations of the absorption study. Formulations containing 50 mM C10 resulted in minimal enterocyte loss and epithelium damage, associated with less FD4 absorption and a shorter absorption window. As the C10 concentration was increased, so did the resulting epithelial damage, extent of FD4 absorption, and the duration of the absorption window. However, already at 60 min, there were signs of recovery, and at 120 min post dose, the intestinal epithelial barrier appeared to be fully recovered, even at the highest evaluated concentration of 300 mM. Although the epithelial barrier had recovered, villus contraction was still observed 120 min after exposure to 300 mM C10. The villus contraction, here seen in response to 100 and 300 mM C10, accelerates the epithelial restitution process by reducing the surface area in need of resealing.^35,36^ The rapid damage to, and recovery of, the intestinal epithelium observed in this study is in agreement with previously published results on the transient effects of C10. Wang et al. administered FD4 by colonic instillation to rats where co-administration of C10 and FD4 resulted in a FD4 bioavailability of 33%.^37^ In contrast, a much lower bioavailability of 8.7 and 4.3% was obtained when C10 was administered 10 and 30 min before FD4, respectively.

The impact of intestinal dilution on absorption of FD4 from C10 formulations was studied by administering a fixed amount of FD4 and C10 in three different volumes. The results showed a trend of decreased bioavailability and *C*_max_ with dilution of the administered formulation. This gives support to the hypothesis that for optimal permeation enhancement, C10 needs to be presented to the intestinal epithelium in a sufficiently high initial concentration and that intestinal dilution is detrimental to the permeation-enhancing effect.^20,23,38^ The two more concentrated administrations (100 and 300 mM C10) achieved similar bioavailability and *C*_max_. This is in contrast to the pH study, where bioavailability increased with the increasing C10 concentration (Table 2), the difference being that the dose increased along with the concentration in the pH study. The group receiving the lowest volume displayed more variable absorption compared to the other groups. The reason for this could be that a smaller volume is more sensitive to intersubject variability in, for instance, the intestinal motility and hence the extent to which the formulation spreads and becomes diluted in the intestine.

The local concentration of C10 and its intestinal dilution are not the only factors important for its permeation-enhancing efficiency. When 16 mg of C10 was instilled in 0.27 mL, resulting in a concentration of 300 mM, the resulting FD4 bioavailability was 22% (Figure 7, Table S1 in the Supporting Information). Increasing the C10 dose three-fold to 47 mg and administering at the same concentration (300 mM), at a volume of 0.8 mL, resulted in a doubling of the bioavailability to 44% (Figure 2D, Table 2). Furthermore, when studying the impact of the colloidal form of C10 on the absorption on FD4, it was observed that the absorption of FD4 increased with the increasing C10 dose (Table 2). Here, the bioavailability of FD4 was 7.9, 21, and 44%, for administrations of 7.8, 16, and 47 mg of C10, respectively. These results indicate that both the local C10 concentration in the intestine and the total dose of C10 administered are important factors for the absorption of FD4.

The C10 concentrations studied in this work were based on the assumption of an enteric-coated dosage form containing 500 mg of C10 (such as GIPET I) dissolving in the small intestine. The highest concentration studied, 300 mM, mimics the scenario where the entire dosage form dissolves in one intestinal fluid pocket (typical volume 4–12 mL^24^). 100 mM C10 simulates the case where drug release occurs across several fluid pockets, and 50 mM C10 corresponds to the case of drug release in the resting small-bowel fluid volume (typical volume 43–105 mL^24^). The permeation-enhancing effect of C10 in this work is similar to that of previously reported studies. Kamio et al. studied FD4 absorption in a rat closed-jejunal loop model where the administration volume was 0.2 mL.^39^ The increase in FD4 AUC compared to controls was 3.4-fold when administered together with 51 mM C10 (10 mg/mL, total C10 dose 2 mg) and 12.6-fold when co-administered with 129 mM C10 (25 mg/mL, 5 mg dose). Maher and colleagues observed a 33-fold increase in FD4 AUC compared to controls when FD4 was instilled with 100 mM C10 to the rat jejunum at an administration volume of 0.2 mL/100 g, corresponding to a C10 dose of 7.8–9.7 mg.^40^ In the present work, the AUC increase compared to controls was 4.9-fold for administrations with 50 mM C10 (7.8 mg C10 dose), 12.9-fold for 100 mM C10 (16 mg C10), and 26.3-fold for administrations with 300 mM C10 (47 mg C10).

Intestinal fluid components, such as bile salts and phospholipids, have been suggested to interact with permeation enhancers, reducing the fraction of the free enhancer available to interact with the intestinal epithelium.^41^ To investigate if bile salts and phospholipids can affect the absorption of FD4 when co-delivered with C10, FD4 and C10 were dissolved in buffer alone or in the simulated intestinal fluids FaSSIF-V2 or FeSSIF-V2. The obtained results were not significantly different, that is, the simulated intestinal fluids did not reduce the absorption of FD4. The results presented here are in contrast to results obtained when other permeation enhancers have been studied. In a study by Gradauer et al. on alkyl-maltosides, the permeation-enhancing effect was abolished in the presence of simulated intestinal fluids.^42^ Another study using the permeation enhancer SNAC also showed lower octreotide apparent permeability in an ex vivo model when formulated in FaSSIF-V2 and rat simulated intestinal fluid, rSIF, compared to when delivered in Krebs–Henseleit buffer.^43^

## Conclusions

In this study, the absorption of FD4, the superficial mucosal injury, and the duration of the window for absorption increased with increasing C10 concentrations with substantial FD4 absorption and concomitant erosion of the enterocyte layer observed at the highest studied C10 concentration of 300 mM. In all cases, however, the intestinal epithelial barrier recovered within 120 min of administration, highlighting the ability of the intestine to remodel and repair itself after exposure to high concentrations of C10. Delivering C10 in its ionized, micellar form at pH 8.5 did not improve the absorption of FD4 at any of the studied concentrations compared to when presented as vesicles at pH 6.5. At the lowest studied C10 concentration of 50 mM, the micellar form performed worse than the vesicular form in terms of enhancing the absorption of FD4, which was associated with a slightly more rapid absorption of C10 for the micellar species.

Increasing the administration volume, thereby simulating greater intestinal dilution of a solid dosage form, resulted in slightly reduced FD4 absorption. In the case of C10, the presence of colloidal structures, here simulated with FaSSIF-V2 and FeSSIF-V2, did not reduce the absorption of FD4 as compared to buffer. Taken together, these findings suggest that when utilizing C10 for enhancing the absorption of macromolecules, both the local concentration and the total dose of the enhancer are of importance for its permeation-enhancing efficacy, whereas the presence of bile-rich colloidal structures does not affect the absorption-enhancing effects of C10.

## Supporting Information Available

Average FD4 plasma concentration–time profiles following IV administration, individual C10 plasma concentration–time profiles, pharmacokinetic parameters for FD4 from intestinal dilution, and vehicle study (PDF)

## Abbreviations

FITC: fluorescein isothiocyanate
FD4: FITC-dextran 4000
C10: sodium caprate

## References

[ref1] Leonard T. W.; et al. Promoting absorption of drugs in humans using medium-chain fatty acid-based solid dosage forms: GIPET. Expert Opin. Drug Deliv. 2006, 3, 685–692. 10.1517/17425247.3.5.685. 16948563

[ref2] Walsh E. G.; et al. Oral delivery of macromolecules: rationale underpinning Gastrointestinal Permeation Enhancement Technology (GIPET). Ther. Deliv. 2011, 2, 1595–1610. 10.4155/tde.11.132. 22833984

[ref3] Amory J. K.; et al. Oral administration of the GnRH antagonist acyline, in a GIPET-enhanced tablet form, acutely suppresses serum testosterone in normal men: single-dose pharmacokinetics and pharmacodynamics. Cancer Chemother. Pharmacol. 2009, 64, 641–645. 10.1007/s00280-009-1038-1. 19479252 PMC2721900

[ref4] Maher S.; et al. Safety and efficacy of sodium caprate in promoting oral drug absorption: from in vitro to the clinic. Adv. Drug Deliv. Rev. 2009, 61, 1427–1449. 10.1016/j.addr.2009.09.006. 19800376

[ref5] Halberg I. B.; et al. The Effect of Food Intake on the Pharmacokinetics of Oral Basal Insulin: A Randomised Crossover Trial in Healthy Male Subjects. Clin. Pharmacokinet. 2019, 58, 1497–1504. 10.1007/s40262-019-00772-2. 31093929 PMC6856260

[ref6] Halberg I. B.; et al. Efficacy and safety of oral basal insulin versus subcutaneous insulin glargine in type 2 diabetes: a randomised, double-blind, phase 2 trial. Lancet 2019, 7, 179–188. 10.1016/s2213-8587(18)30372-3. 30679095

[ref7] Tillman L. G.; Geary R. S.; Hardee G. E. Oral delivery of antisense oligonucleotides in man. J. Pharm. Sci. 2008, 97, 225–236. 10.1002/jps.21084. 17721945

[ref8] Gennemark P.; et al. An oral antisense oligonucleotide for PCSK9 inhibition. Sci. Transl. Med. 2021, 13, eabe9117 10.1126/scitranslmed.abe9117. 33980578

[ref9] Kanicky J. R.; Shah D. O. Effect of Premicellar Aggregation on the p*K*_a_ of Fatty Acid Soap Solutions. Langmuir 2003, 19, 2034–2038. 10.1021/la020672y.

[ref10] Namani T.; Walde P. From decanoate micelles to decanoic acid/dodecylbenzenesulfonate vesicles. Langmuir 2005, 21, 6210–6219. 10.1021/la047028z. 15982022

[ref11] Morigaki K.; et al. Thermodynamic and kinetic stability. Properties of micelles and vesicles formed by the decanoic acid/decanoate system. Colloids Surf., A 2003, 213, 37–44. 10.1016/s0927-7757(02)00336-9.

[ref12] Kronberg B.; Holmberg K.; Lindman B.; Two Fundamental Forces in Surface and Colloid Chemistry. In Surface Chemistry of Surfactants and Polymers, Kronberg B.; Holmberg K., Eds.; John Wiley & Sons, Ltd., 2014; pp 65–74.

[ref13] Twarog C.; et al. A head-to-head Caco-2 assay comparison of the mechanisms of action of the intestinal permeation enhancers: SNAC and sodium caprate (C10). Eur. J. Pharm. Biopharm. 2020, 152, 95–107. 10.1016/j.ejpb.2020.04.023. 32387703

[ref14] Krug S. M.; et al. Sodium caprate as an enhancer of macromolecule permeation across tricellular tight junctions of intestinal cells. Biomaterials 2013, 34, 275–282. 10.1016/j.biomaterials.2012.09.051. 23069717

[ref15] Anderberg E. K.; Lindmark T.; Artursson P. Sodium caprate elicits dilatations in human intestinal tight junctions and enhances drug absorption by the paracellular route. Pharm. Res. 1993, 10, 857–864. 10.1023/a:1018909210879. 8321854

[ref16] Lindmark T.; Nikkilä T.; Artursson P. Mechanisms of absorption enhancement by medium chain fatty acids in intestinal epithelial Caco-2 cell monolayers. J. Pharmacol. Exp. Ther. 1995, 275, 958–964. 7473188

[ref17] Lindmark T.; Kimura Y.; Artursson P. Absorption enhancement through intracellular regulation of tight junction permeability by medium chain fatty acids in Caco-2 cells. J. Pharmacol. Exp. Ther. 1998, 284, 362–369. 9435199

[ref18] Tomita M.; Hayashi M.; Awazu S. Absorption-enhancing mechanism of sodium caprate and decanoylcarnitine in Caco-2 cells. J. Pharmacol. Exp. Ther. 1995, 272, 739–743. 7853188

[ref19] Brayden D. J.; Gleeson J.; Walsh E. G. A head-to-head multi-parametric high content analysis of a series of medium chain fatty acid intestinal permeation enhancers in Caco-2 cells. Eur. J. Pharm. Biopharm. 2014, 88, 830–839. 10.1016/j.ejpb.2014.10.008. 25460147

[ref20] Maher S.; et al. Application of Permeation Enhancers in Oral Delivery of Macromolecules: An Update. Pharmaceutics 2019, 11, 41 10.3390/pharmaceutics11010041. PMC6359609 30669434

[ref21] Maher S.; et al. Effects of surfactant-based permeation enhancers on mannitol permeability, histology, and electrogenic ion transport responses in excised rat colonic mucosae. Int. J. Pharm. 2018, 539, 11–22. 10.1016/j.ijpharm.2018.01.008. 29341916

[ref22] Twarog C.; et al. Intestinal Permeation Enhancers for Oral Delivery of Macromolecules: A Comparison between Salcaprozate Sodium (SNAC) and Sodium Caprate (C10). Pharmaceutics 2019, 11, 78 10.3390/pharmaceutics11020078. PMC6410172 30781867

[ref23] Maher S.; Geoghegan C.; Brayden D. J. Intestinal permeation enhancers to improve oral bioavailability of macromolecules: reasons for low efficacy in humans. Expert Opin. Drug Deliv. 2020, 18, 273–300. 10.1080/17425247.2021.1825375. 32937089

[ref24] Vinarov Z.; et al. Impact of gastrointestinal tract variability on oral drug absorption and pharmacokinetics: an UNGAP review. Eur. J. Pharm. Sci. 2021, 162, 105812 10.1016/j.ejps.2021.105812. 33753215

[ref25] Almgren M.; Edwards K.; Karlsson G. Cryo transmission electron microscopy of liposomes and related structures. Colloids Surf., A 2000, 174, 3–21. 10.1016/s0927-7757(00)00516-1.

[ref26] Langenbucher F. Numerical convolution/deconvolution as a tool for correlating in vitro with in vivo drug availability. Pharm. Ind. 1982, 44, 1166–1172.

[ref27] Sjögren E.; et al. In silico predictions of gastrointestinal drug absorption in pharmaceutical product development: application of the mechanistic absorption model GI-Sim. Eur. J. Pharm. Sci. 2013, 49, 679–698. 10.1016/j.ejps.2013.05.019. 23727464

[ref28] Sjögren E.; Thörn H.; Tannergren C. In Silico Modeling of Gastrointestinal Drug Absorption: Predictive Performance of Three Physiologically Based Absorption Models. Mol. Pharm. 2016, 13, 1763–1778. 10.1021/acs.molpharmaceut.5b00861. 26926043

[ref29] Ahmad A.; et al. IMI – Oral biopharmaceutics tools project – Evaluation of bottom-up PBPK prediction success part 4: Prediction accuracy and software comparisons with improved data and modelling strategies. Eur. J. Pharm. Biopharm. 2020, 156, 50 10.1016/j.ejpb.2020.08.006. 32805361

[ref30] Mehvar R.; Shepard T. L. Molecular-weight-dependent pharmacokinetics of fluorescein-labeled dextrans in rats. J. Pharm. Sci. 1992, 81, 908–912. 10.1002/jps.2600810914. 1279158

[ref31] Kararli T. T. Comparison of the gastrointestinal anatomy, physiology, and biochemistry of humans and commonly used laboratory animals. Biopharm. Drug Dispos. 1995, 16, 351–380. 10.1002/bdd.2510160502. 8527686

[ref32] McConnell E. L.; Basit A. W.; Murdan S. Measurements of rat and mouse gastrointestinal pH, fluid and lymphoid tissue, and implications for in vivo experiments. J. Pharm. Pharmacol. 2008, 60, 63–70. 10.1211/jpp.60.1.0008. 18088506

[ref33] Tanaka Y.; et al. Regional differences in the components of luminal water from rat gastrointestinal tract and comparison with other species. J. Pharm. Pharm. Sci. 2012, 15, 510–518. 10.18433/j3f602. 23106954

[ref34] Christfort J. F.; et al. Developing a predictive in vitro dissolution model based on gastrointestinal fluid characterisation in rats. Eur. J. Pharm. Biopharm. 2019, 142, 307–314. 10.1016/j.ejpb.2019.07.007. 31288077

[ref35] Moore R.; Carlson S.; Madara J. L. Villus contraction aids repair of intestinal epithelium after injury. Am. J. Physiol. 1989, 257, G274–G283. 10.1152/ajpgi.1989.257.2.g274. 2764111

[ref36] Blikslager A. T.; et al. Restoration of Barrier Function in Injured Intestinal Mucosa. Physiol. Rev. 2007, 87, 545–564. 10.1152/physrev.00012.2006. 17429041

[ref37] Wang X.; Maher S.; Brayden D. J. Restoration of rat colonic epithelium after in situ intestinal instillation of the absorption promoter, sodium caprate. Ther. Deliv. 2010, 1, 75–82. 10.4155/tde.10.5. 22816121

[ref38] Maher S.; Brayden D. J. Formulation strategies to improve the efficacy of intestinal permeation enhancers,. Adv. Drug Delivery Rev. 2021, 177, 113925 10.1016/j.addr.2021.113925. 34418495

[ref39] Kamio Y.; et al. Epinephrine is an enhancer of rat intestinal absorption. J. Controlled Release 2005, 102, 563–568. 10.1016/j.jconrel.2004.11.003. 15681079

[ref40] Maher S.; et al. Evaluation of intestinal absorption and mucosal toxicity using two promoters. II. Rat instillation and perfusion studies. Eur. J. Pharm. Sci. 2009, 38, 301–311. 10.1016/j.ejps.2009.07.011. 19664704

[ref41] Hossain S.; et al. Influence of Bile Composition on Membrane Incorporation of Transient Permeability Enhancers. Mol. Pharm. 2020, 17, 4226 10.1021/acs.molpharmaceut.0c00668. 32960068 PMC7610231

[ref42] Gradauer K.; et al. Interaction with Mixed Micelles in the Intestine Attenuates the Permeation Enhancing Potential of Alkyl-Maltosides. Mol. Pharm. 2015, 12, 2245–2253. 10.1021/mp500776a. 25874852

[ref43] Fattah S.; et al. Salcaprozate sodium (SNAC) enhances permeability of octreotide across isolated rat and human intestinal epithelial mucosae in Ussing chambers. Eur. J. Pharm. Sci. 2020, 154, 105509 10.1016/j.ejps.2020.105509. 32777258

