# Supplementary material for: Impact of Intestinal Concentration and Colloidal Structure on the Permeation-Enhancing Efficiency of Sodium Caprate in the Rat
Source: Mol Pharm. 2021 Dec 20;19(1):200–12. doi: 10.1021/acs.molpharmaceut.1c00724 (PMC8728734; doi:10.1021/acs.molpharmaceut.1c00724)
Supplement: Supplementary file 1 — mp1c00724_si_001.pdf [file mp1c00724_si_001.pdf]

Supporting information for

Impact of intestinal concentration and colloidal structure on the permeation enhancing efficiency of sodium caprate in the rat

Staffan Berg[a,b], Lillevi Kärrberg[c], Denny Suljovic[a], Frank Seeliger[d], Magnus Söderberg[d],

Marta Perez-Alcazar[e], Natalie Van Zuydam[f], Bertil Abrahamsson[g], Andreas Martin Hugerth[h],

Nigel Davies[b], Christel A.S. Bergström[a]

[a] The Swedish Drug delivery Center, Department of Pharmacy, Uppsala University, BMC P.O. Box 580, SE-751 23 Uppsala, Sweden
[b] Advanced Drug Delivery, Pharmaceutical Sciences, R&D, AstraZeneca, Gothenburg, Sweden
[c] Animal Sciences and Technologies, Clinical Pharmacology and Safety Sciences, Biopharmaceuticals R&D, AstraZeneca, Gothenburg, Sweden
[d] Cardiovascular, Renal and Metabolism Safety, Clinical Pharmacology & Safety Sciences, BioPharmaceuticals R&D, AstraZeneca, Gothenburg, Sweden
[e] Imaging and Data Analytics, Clinical Pharmacology and Safety Sciences, Biopharmaceuticals R&D, AstraZeneca, Gothenburg, Sweden
[f] Data Science and Quantitative Biology, Discovery Sciences, BioPharmaceuticals R&D, AstraZeneca, Gothenburg, Sweden
[g] Oral Product Development, Pharmaceutical Technology & Development, Operations, AstraZeneca Gothenburg, Sweden
[g] Ferring Pharmaceuticals A/S Global Pharmaceutical R&D, Copenhagen, Denmark

Table of content

Figure S1. Average FD4 plasma concentration-time profiles after intravenous bolus administration to rats.

Figure S2. Individual C10 plasma concentration-time profiles after intraduodenal bolus administrations in rats.

Table S1. Effect of intestinal dilution.

Table S2. Effect of vehicle composition.

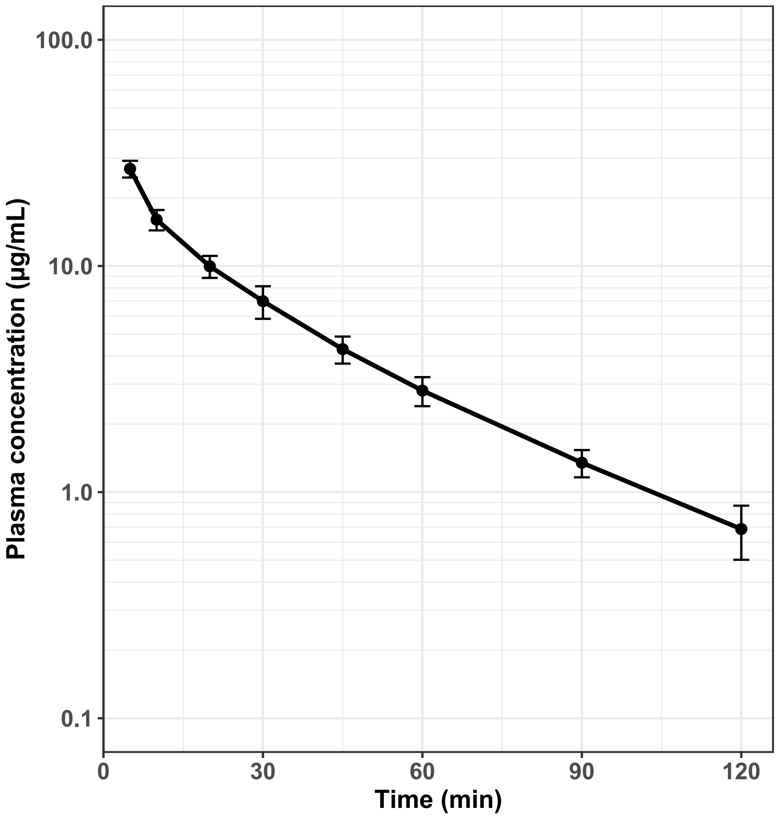

Figure S1. The average (±SD) FD4 plasma concentration-time profiles after intravenous bolus administrations of 5 mg/kg to anesthetized rats (n=6).

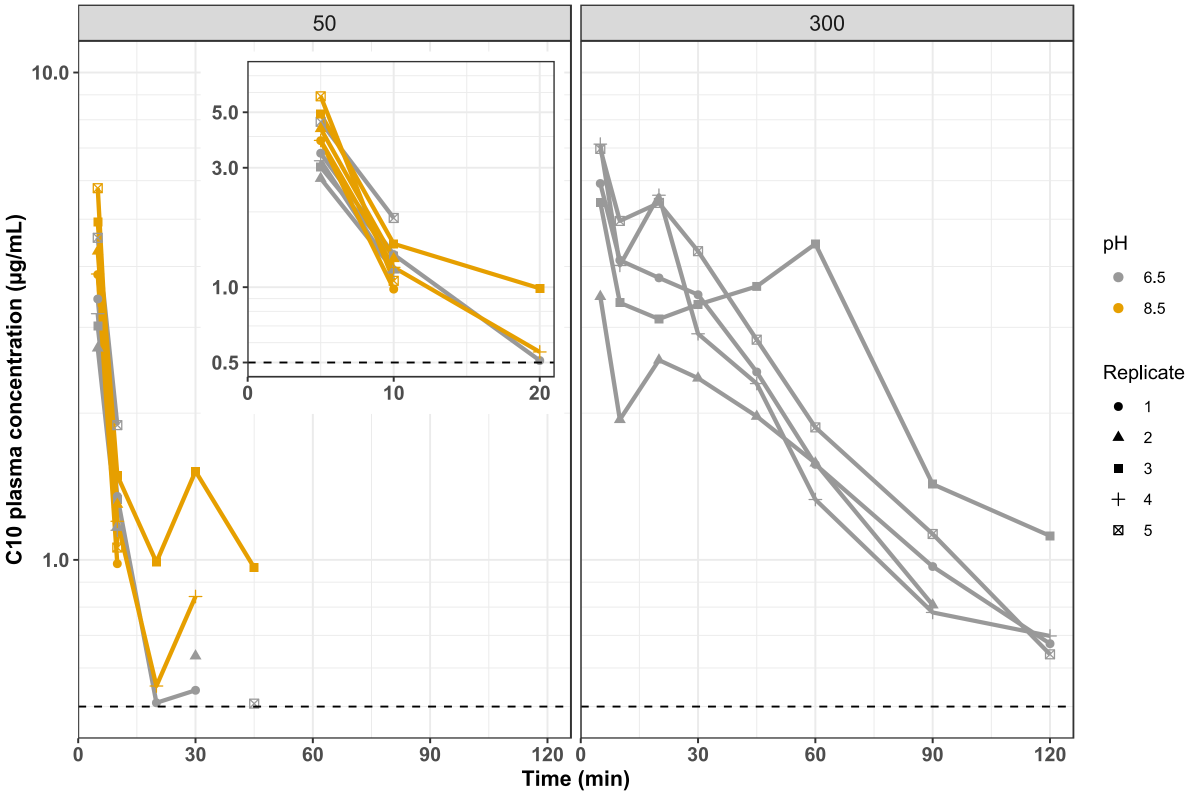

Figure S2. Individual C10 plasma concentration-time profiles after intraduodenal bolus administrations to anesthetized rats (n=5 /group). The formulations contained 50 mM C10 pH 6.5, 50 mM C10 pH 8.5, and 300 mM C10 pH 6.5 in blank FaSSIF. The dashed line indicates the lower limit of quantification.

Table S1. Effect of intestinal dilution. Pharmacokinetic parameters for FD4 following duodenal administration of 10 mg FD4 and 16 mg C10 in different dose volumes to anesthetized rats. All formulations were prepared in blank FaSSIF and adjusted to pH 6.5.

**Dose volume, mL**	**AUC_{0-120}, min*µg/mL**	**F, %**	**C_{max}, µg/mL**	**t_{max}, min**
0.27	1120 ±390 (35)	22 ±8.1 (37)	20 ±6.4 (32)	10 (10/20)
0.8	1070 ±160 (15)	21 ±3.3 (16)	18 ±1.3 (7.4)	10 (10/10)
1.6	703 ±67 (9.6)	14 ±1.3 (9.4)	15 ±2.2 (15)	5 (5/10)

AUC, F and C_{max} are reported as mean ±SD (CV %), t_{max} as median (min/max), n=5 /group.

Table S2. Effect of vehicle composition. Pharmacokinetic parameters for FD4 following intraduodenal administration to rats. Formulations were prepared in buffer (blank FaSSIF) or the simulated intestinal fluids FaSSIF-V2 and FeSSIF-V2 and adjusted to pH 6.5.

**C10 conc., mM**	**Buffer**	**AUC_{0-120}, min*µg/mL**	**F, %**	**C_{max}, µg/mL**	**t_{max}, min**
50	Blank FaSSIF	405 ±60 (15)	7.9 ±1.2 (15)	9.5 ±1.6 (17)	10 (5/10)
50	FaSSIF-V2	578 ±170 (30)	11 ±3.3 (29)	14 ±4.0 (29)	5 (5/10)
50	FeSSIF-V2	579 ±160 (27)	12 ±4.0 (34)	12 ±3.2 (25)	10 (5/10)
300	Blank FaSSIF	2180 ±540 (25)	44 ±11 (26)	28 ±6.7 (23)	30 (30/45)
300	FaSSIF-V2	2400 ±380 (16)	47 ±7.5 (16)	32 ±8.5 (27)	30 (20/45)
300	FeSSIF-V2	2490 ±400 (16)	49 ±7.9 (16)	32 ±4.9 (15)	30 (20/30)

AUC, F and C_{max} are reported as mean ±SD (CV %), t_{max} as median (min/max), n=5 /group.
